# Whey Improves In Vitro Endothelial Mitochondrial Function and Metabolic Redox Status in Diabetic State

**DOI:** 10.3390/antiox12061311

**Published:** 2023-06-20

**Authors:** Elisa Martino, Amalia Luce, Anna Balestrieri, Luigi Mele, Camilla Anastasio, Nunzia D’Onofrio, Maria Luisa Balestrieri, Giuseppe Campanile

**Affiliations:** 1 Department of Precision Medicine, University of Campania Luigi Vanvitelli, Via L. De Crecchio 7, 80138 Naples, Italy; elisa.martino@unicampania.it (E.M.); amalia.luce@unicampania.it (A.L.); camilla.anastasio@unicampania.it (C.A.); nunzia.donofrio@unicampania.it (N.D.); 2 Food Safety Department, Istituto Zooprofilattico Sperimentale del Mezzogiorno, 80055 Portici, Italy; anna.balestrieri@izsmportici.it; 3 Department of Experimental Medicine, University of Campania Luigi Vanvitelli, Via Luciano Armanni 5, 80138 Naples, Italy; luigi.mele@unicampania.it; 4 Department of Veterinary Medicine and Animal Production, University of Naples Federico II, 80137 Naples, Italy; giuseppe.campanile@unina.it

**Keywords:** endothelial dysfunction, diabetes, obesity, metabolism, mitochondria, whey, SIRT3

## Abstract

Endothelial dysfunction plays a critical role in the progression of type 2 diabetes mellitus (T2DM), leading to cardiovascular complications. Current preventive antioxidant strategies to reduce oxidative stress and improve mitochondrial function in T2DM highlight dietary interventions as a promising approach, stimulating the deepening of knowledge of food sources rich in bioactive components. Whey (WH), a dairy by-product with a considerable content of bioactive compounds (betaines and acylcarnitines), modulates cancer cell metabolism by acting on mitochondrial energy metabolism. Here, we aimed at covering the lack of knowledge on the possible effect of WH on the mitochondrial function in T2DM. The results showed that WH improved human endothelial cell (TeloHAEC) function during the in vitro diabetic condition mimicked by treating cells with palmitic acid (PA) (0.1 mM) and high glucose (HG) (30 mM). Of note, WH protected endothelial cells from PA+HG-induced cytotoxicity (*p* < 0.01) and prevented cell cycle arrest, apoptotic cell death, redox imbalance, and metabolic alteration (*p* < 0.01). Moreover, WH counteracted mitochondrial injury and restored SIRT3 levels (*p* < 0.01). The SiRNA-mediated suppression of SIRT3 abolished the protective effects exerted by WH on the mitochondrial and metabolic impairment caused by PA+HG. These in vitro results reveal the efficacy of whey as a redox and metabolic modulator in the diabetic state and pave the way for future studies to consider whey as the source of dietary bioactive molecules with health benefits in preventive strategies against chronic diseases.

## 1. Introduction

Endothelial dysfunction and epigenetic modification of endothelium represent a hallmark of many human diseases, including atherosclerosis, hypertension, and diabetes [1,2]. Type 2 diabetes mellitus (T2DM) is an expanding global health disorder, with increasing prevalence in the younger population, that requires continuous new knowledge for the reduction in risk factors involved in the etiopathogenesis of the disease [3,4]. The development and progression of T2DM include several mechanisms, such as chronic low-grade inflammation, hyperglycemia, insulin secretion deficiency, autophagy dysregulation, electrolyte imbalance, oxidative stress, and disruption of endothelial function [5,6]. Developing effective methods to prevent or retard the progression of T2DM associated with oxidative stress is still compelling [7,8]. Endothelial injury in diabetic conditions is linked to mitochondrial dysfunction, leading to decreased mitochondrial membrane potential and damaged mitochondrial respiration, as well as excessive production of mitochondrial reactive oxygen species (ROS) [5,9,10]. These phenomena are responsible for impaired ATP synthesis, apoptosis, and activation of downstream inflammatory signaling pathways [5,9,10]. In endothelial cells (EC), harmful ROS are inactivated by sirtuin 3 (SIRT3), the mitochondrial NAD^+^-dependent deacetylase which emerged as an important contributor to the endothelial energy homeostasis [11,12]. Redox changes at the mitochondrial level and deficiency of SIRT3 have been associated with metabolic pathologies and endothelial dysfunction, thus increasing the interest toward the identification of mitochondrial targets and their involvement in chronic diseases [13,14]. Although the remarkable contribution of genetic factors in the pathogenesis of T2DM is well established, the constant incidence increase in diabetic patients targets focal attention on environmental determinants like modifiable risk factors, such as obesity, physical inactivity, and dietary habits. Epidemiological studies reported that adequate dairy intake may lower the incidence of cardiovascular diseases, underlining that acting on modifiable risk factors for T2DM development could be effective in the prevention of disease [15,16,17]. To this aim, the search for nutrients and food-derived compounds able to modulate age-related epigenetic mechanisms promoting beneficial/preventive effects still represents an open field. The latest findings show that improved mitochondrial redox capacity by dietary interventions may be an important strategy for preventing and/or treating T2DM and its complications [18,19,20,21,22]. In this regard, the ability of milk to exert positive effects on human health can be ascribed to its distinguishing content in functional compounds and exosome microRNAs (miRNAs), mainly from Italian Mediterranean buffalo (*Bubalus bubalis*) [23,24,25,26,27,28]. Recent studies described the antiproliferative effects of milk, milk-derived bioactive compounds, exosomes, and vesicle-derived miRNAs in different human cancer cells [29,30,31,32,33,34,35], as well as their ability to protect EC by chronic dysfunction [13,28,36]. In addition to milk, whey (WH) obtained from white cheese production still maintains a notable content in betaines, l-carnitine, and acylcarnitines [37,38]. Previous evidence described the pharmacological ability of WH to ameliorate energetic homeostasis and muscle mass rate in cancer patients [39,40]. In vitro and in vivo studies proved its anticancer activities [38,41]. Indeed, WH induced cell cycle arrest and apoptosis via caspase-3 activation by limiting glucose uptake and interfering with mitochondrial energy metabolism via sirtuin(s) modulation in different colorectal cell lines [41], while it sustained necroptotic death and metabolic stress in xenograft mice [38]. Nevertheless, the protective effect of WH against T2DM-related dysfunctional endothelium has not been evaluated.

In light of this evidence, here, we aimed at investigating the effect of WH on the metabolic and mitochondrial redox changes in diabetic EC to evaluate its potential role as a preventive dietary source for a healthy and functional EC phenotype to reduce the burden of T2DM.

## 2. Materials and Methods

### 2.1. WH Extraction and Metabolic Characterization

WH collection and extraction were carried out as previously described [35,38]. WH of Italian Mediterranean buffalo (*Bubalus bubalis*) milk was defatted by centrifugating at 3000× *g* for 15 min, casein precipitated by adjusting the pH to 4.6 with hydrochloric acid, and, after 1 h centrifugation at 10,000× *g*, the supernatant was filtered by 3-kDa Amicon ultra centrifugal filters to recover low molecular weight metabolites. The metabolic profile was assessed by HPLC-ESI-MS/MS analyses with Agilent LC-MSD SL quadrupole ion trap in positive multiple reaction monitoring (MRM), as already reported [26,27,31]. Chromatography was performed with 0.1% formic acid in water in isocratic mode at a flow rate of 100 µL/min. Standard and samples were injected (5 µL) by autosampler and compounds characterized according to specific retention times and MS2 fragmentation patterns. The content of each biomolecule, reported as mg/L, was determined by comparing the peak area of the highest MS2 fragment with standard calibration curve. Analyses were conducted in triplicate and linearity derived by correlation coefficients (r^2^) > 0.99. For cell treatments, sterilization of WH extract was carried out by 0.22 µm Millipore filter. 

### 2.2. Cell Line and Culture Conditions

TeloHAEC (CRL-4052) cultured in vascular cell basal media (PCS-100-030) supplemented with cell growth kit-VEGF (PCS-100-041) were all obtained from the American Type Culture Collection (ATCC, Manassas, VA, USA) and properly maintained at 5% CO_2_ in 37 °C incubator. The concomitant stimulation with high glucose (HG) and fatty acid (palmitic acid, PA) was used to mimic the in vivo T2DM-related hyperglycemia and hyperlipidemia condition on EC, as reported in different in vitro T2DM models [42,43,44]. EC were treated with PA (up to 0.5 mM) and HG (up to 30 mM) for a maximum time of 72 h. WH treatments were performed up to 72 h with different volumes (5–20% *v*/*v*). For combined treatments, EC were treated for 48 h with 0.1 mM PA and 30 mM HG supplemented with WH (20% *v*/*v*) (WH+PA+HG). For mechanistic studies, EC were transfected with Lipofectamin (Vehicle) by using 30 nM SIRT3 siRNA (438080910101, Applied Biological Materials, Inc., Richmond, BC, Canada) or nontargeting siRNA control (NT) in serum-free medium. At 24 h after transfection, the above-described treatments were performed.

### 2.3. Viability and Cytotoxicity

EC viability assay was conducted according to the manufacturer’s instructions (CCK-8 CK04, Donjindo Molecular Technologies, Inc., Rockville, MD, USA). Experiments were initiated with addition of Kit-8 solution in quadruplicates, followed by incubation for 4 h. Cytotoxicity assay (CK12, Donjindo Molecular Technologies, Inc., Rockville, MD, USA) was used to evaluate the LDH release in the media based on the instructions of the manufacturer. A Bio-Rad model 680 (Bio-Rad, Hercules, CA, USA) microplate reader was used to take readings at 450 and 490 nm, respectively. 

### 2.4. HDAC3 and SIRT3 Assays

Histone Deacetylase 3 (HDAC3) activity assay kit (EPI004, Sigma-Aldrich, St. Louis, MO, USA) and Sirtuin 3 ELISA Kit (MBS3803577, MyBioSource, San Diego, CA, USA) were used, according to the manufacturer’s instructions, to assess HDAC3 levels in cell lysates and SIRT3 content in culture medium, respectively. Fluorescence (Ex/Em = 380/500 nm) and absorbance (450 nm) values were recorded using Tecan Infinite 2000 (Tecan, Männedorf, Switzerland) and Bio-Rad 680 (Bio-Rad, Hercules, CA, USA) plate readers. Experiments were performed with *n* = 4 replicates.

### 2.5. Cell Cycle and Death 

For cell cycle experiments, synchronized EC by serum-starved were seeded, before performing the different treatments in complete culture media. Cell cycle was evaluated by incubating for 30 min trypsinized EC with PI-stain buffer, whilst apoptosis was detected with FITC Annexin V Apoptosis Detection Kit (556547, BD Pharmigen, Franklin Lakes, NJ, USA), as previously described [14]. Both assays were recorded by a BD Accuri C6 cytometer (BD Biosciences, San José, CA, USA) and analyses performed with FlowJo V10 software (Williamson Way, Ashland, OR, USA). For cell death analysis, the staining of cells simultaneously with Annexin V and PI allowed for the discrimination of viable cells (negative for both Annexin V and PI), early apoptotic (positive only for Annexin V), late apoptotic (positive for both Annexin V and PI), and necrotic cells (positive only for PI).

### 2.6. Oxidative State 

MDA assay kit (ab118970, Abcam, Cambridge, UK), Lactate Assay Kit-WST (L256, Dojindo Molecular Technologies, Tokyo, Japan), NAD/NADH Assay Kit-WST (N509, Dojindo Molecular Technologies, Tokyo, Japan), and GSSG/GSH quantification kit (G257, Dojindo Molecular Technologies, Tokyo, Japan) were conducted according to the supplier’s instructions, in order to evaluate the EC oxidative state. The specific absorbance was measured using a Bio-Rad 680 microplate reader (Bio-Rad, Hercules, CA, USA). Experiments were performed with *n* = 4 replicates.

### 2.7. Reactive Oxygen Species (ROS)

Intracellular and mitochondrial ROS levels were assessed by CellROX Green (C10444, Invitrogen, Waltham, MA, USA) and MitoSOX Red (M36008, Invitrogen, Waltham, MA, USA) probes, according to the manufacturer’s instructions. Fluorescent images were acquired by EVOS M5000 microscope (Thermo Scientific, Rockford, IL, USA) and fluorescence recorded by a BD Accuri C6 cytometer (BD Biosciences, San José, CA, USA). Extracellular ROS were determined with Amplex Red Hydrogen Peroxide/Peroxidase Assay kit (A22188, Invitrogen, Waltham, MA, USA), according to the supplier’s instructions, and fluorescence (Ex/Em = 530/590 nm) recorded using a Tecan Infinite 2000 (Tecan, Männedorf, Switzerland) plate reader. Experiments were performed with *n* = 4 replicates.

### 2.8. Mitochondrial Function 

Mitophagy detection kit (MD01, Dojindo Molecular Technologies, Tokyo, Japan) and mitochondrial membrane potential probe tetraethylbenzimidazolylcarbocyanine iodide (JC-1) (MT09, Dojindo Molecular Technologies, Tokyo, Japan) stains were carried out as previously reported [14]. Fluorescent images were recorded by EVOS M5000 microscope (Thermo Scientific, Rockford, IL, USA) while fluorescence intensities detected by a BD Accuri C6 cytometer (BD Biosciences, San José, CA, USA). Analyses were performed with FlowJo V10 software (Williamson Way, Ashland, OR, USA). The ATP kit (ab83355, Abcam, Cambridge, UK) was used, according to the supplier’s instructions, to determine the ATP content. The 570 nm absorbance was measured by a Bio-Rad 680 microplate reader (Bio-Rad, Hercules, CA, USA). Experiments were performed with *n* = 4 replicates.

### 2.9. Mitochondrial Respiration

The mitochondrial respiration was assessed with the Seahorse MitoStress kit (103010-100, Agilent), following the manufacturer’s instructions. EC were seeded in Seahorse assay microplate and grown in 100 μL of growth medium or treatments prior to analysis. The day before the assay, a cartridge was hydrated using an XF calibrant (103059-000, Agilent Technologies, Santa Clara, CA, USA) and incubated overnight at 37 °C in a non-CO_2_ incubator. Prior to the assay, culture media were changed to 180 µL/well XF DMEM supplemented with 10 mM glucose, 2 mM glutamine, and 1 mM pyruvate (103575-100, 103577-100, 103579-100, 103578-100, all from Agilent Technologies, Santa Clara, CA, USA) and plate incubated for 1 h at 37 °C (without CO_2_). The oxygen consumption rate (OCR) and the extracellular acidification rate (ECAR) were determined on an XF HS Seahorse Bioanalyzer (Agilent Technologies, Santa Clara, CA, USA). The final concentration of injected compounds was 1.5 μM oligomycin, 1 μM carbonyl cyanide-4(trifluoromethoxy) phenylhydrazone (FCCP), and 0.5 μM rotenone + antimycin A. Data were normalized by the protein concentration of its corresponding well.

### 2.10. Western Blotting

EC were lysed as already described [14]. Homogenates were resolved with 8–10% SDS-PAGE electrophoresis, transferred to nitrocellulose membranes, and blocked with 5% 1X Tris-Buffered Saline (TBS) 1% casein blocker (1610782, Bio-Rad, Hercules, CA, USA). Then, the membranes were immunodetected with primary antibodies against SIRT3 (1:2000, PA5-86035, Invitrogen, Waltham, MA, USA), BAX (1:500, orb216030, Biorbyt, Cambridge, UK), Bcl-2 (1:500, E-AB-15522, Elabscience Biotechnology Inc., Houston, TX, USA), COX-IV (1:2000, MA5-15078, Invitrogen, Waltham, MA, USA), caspase-3 (1:1000, 9662, Cell Signaling Technology, Danvers, MA, USA), caspase-9 (1:1000, 9508, Cell Signaling Technology, Danvers, MA, USA), PARP (1:1000, ab194586, Abcam, Cambridge, UK), cyclin D1 (1:1000, ab134175, Abcam, Cambridge, UK), cyclin E1 (1:1000, ab33911, Abcam, Cambridge, UK), SREBP1 (1:1000, Abcam, ab28481, Cambridge, UK), PPAR-α (1:1000, Elabscience Biotechnology Inc., Houston, TX, USA, E-AB-32646), PPAR-γ (1:1000, Biorbyt, orb69095, Cambridge, UK), LDHA (1:1000, ThermoFisher Scientific, PA5-27406, Waltham, MA, USA), α-tubulin (1:5000, E-AB-20036, Elabscience Biotechnology Inc., Houston, TX, USA), actin (1:3000, ab179467, Abcam, Cambridge, UK), and GAPDH (1:2000, ab9485, Abcam, Cambridge, UK). After 1 h incubation with HRP-labeled secondary antibody at room temperature, the ECL kit (E-IR-R301, Elabscience Biotechnology Inc., Houston, TX, USA) was applied on dried membranes and chemiluminescence detected by ChemiDoc Imaging System with Image Lab 6.0.1 software (Bio-Rad Laboratories, Milan, Italy). The density of protein bands was analyzed by ImageJ 1.52n version software (Wayne Rasband, National Institutes of Health, Bethesda, MD, USA) and then normalized to the relative loading control.

### 2.11. Statistical Analysis

GraphPad Prism version 9.1.2 software (Software Inc., La Jolla, CA, USA) was used to conduct all statistical analyses. Statistical significance, determined using multiple *t* tests, was set at a *p*-value < 0.05. The results were calculated in means ± standard deviations (SD).

## 3. Results

### 3.1. WH Characterization 

The metabolic profile of WH was evaluated by HPLC-ESI-MS/MS analysis. In line with previous studies [37], the 3-kDa WH extract showed a considerable content of betaines and short-chain acylcarnitines as follows: 43.4 ± 1.6 mg/L l-carnitine, 46.5 ± 3.1 mg/L acetyl-l-carnitine, 29.4 ± 2.2 mg/L propionyl-l-carnitine, 13.2 ± 1.2 mg/L glycine betaine, 26.7 ± 2.3 mg/L δ-valerobetaine, and 6.8 ± 0.9 mg/L γ-butyrobetaine (Table 1).

### 3.2. In Vitro T2DM and Cell Cycle Analysis

**Figure 1:**
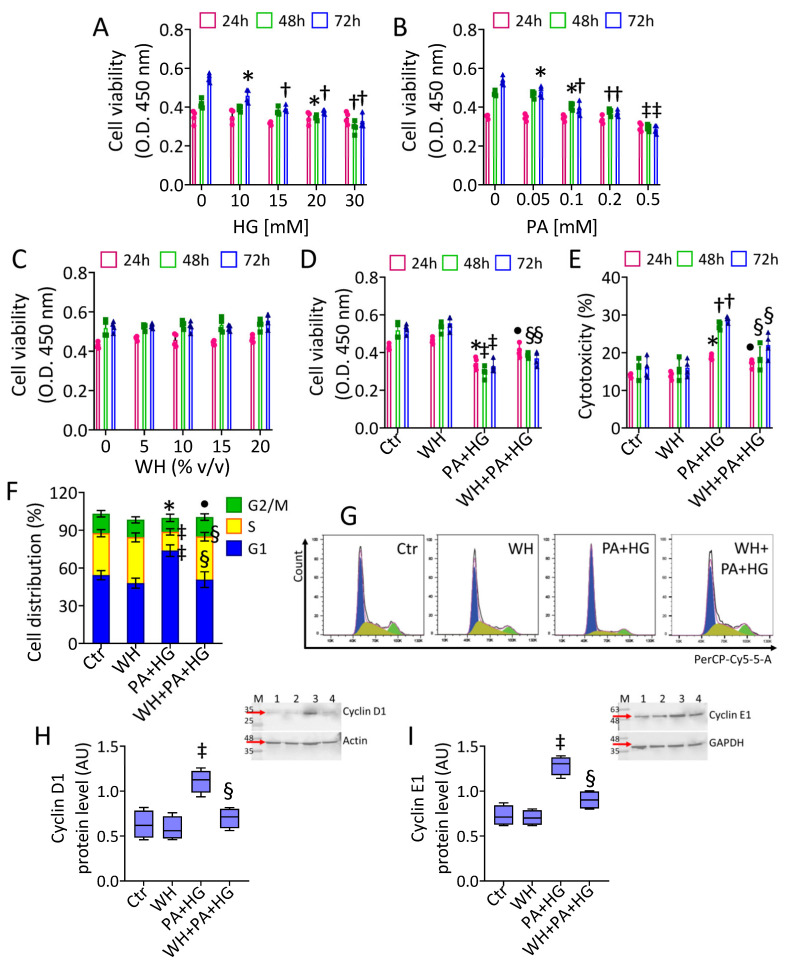
Whey effects on PA+HG-perturbated cell cycle. EC viability following exposure for different times and doses to (**A**) glucose (HG), (**B**) palmitic acid (PA), and (**C**) whey (WH). (**D**) Viability and (**E**) cytotoxicity evaluated on EC after 48 h exposure to PA+HG (0.1 mM and 30 mM, respectively) with or without WH supplementation (20% *v*/*v*). (**F**,**G**) Representative cell cycle detection by cytometric analysis and immunoblotting analysis with cropped blots of (**H**) cyclin D1 and (**I**) cyclin E1. Data are reported as mean ± SD of n = 3 independent experiments. M = molecular weight markers, lane 1 = Ctr, lane 2 = WH, Lane 3 = PA+HG, lane 4 = WH+PA+HG. Western blotting results are expressed as arbitrary units (AU). * *p* < 0.05 vs. 0 or Ctr; † *p* < 0.01 vs. 0 or Ctr; ‡ *p* < 0.001 vs. 0 or Ctr; • *p* < 0.05 vs. PA+HG; § *p* < 0.01 vs. PA+HG.

To investigate the effect of WH on T2DM-mediated dysfunctional endothelium, an in vitro model of TeloHAEC cells treated with combined HG and PA treatment was used [42,43,44]. The single effect of different concentrations of HG (10, 15, 20, and 30 mM) and PA (0.05, 0.1, 0.2, and 0.5 mM) on cell viability was investigated. The results showed that 30 mM HG (*p* < 0.01) and 0.5 mM PA (*p* < 0.001) induced the highest cytotoxic effects after 48 h, maintaining this similar trend at 72 h (Figure 1A,B).

Then, increasing PA concentrations up to 72 h were added to 30 mM HG cells (Figure S1). Dose-response experiments indicated that 0.1 mM PA was the lowest effective concentration in aggravating the HG-related cytotoxicity (*p* < 0.05 vs. HG) (Figure S1). Therefore, 48 h exposure to 0.1 mM PA and 30 mM HG (PA+HG) was used for the subsequent experiments. We next evaluated the effect of 3-kDa WH in the presence of PA+HG stress. The results showed that EC viability was not affected by treatment with 3-kDa WH alone up to 72 h of incubation with 20% (*v*/*v*) (Figure 1C). Of note, supplementation with 20% (*v*/*v*) 3-kDa WH prevented the 48 h PA+HG-induced damage, as assessed by both viability and cytotoxicity evaluation (*p* < 0.01 vs. PA+HG) (Figure 1D,E). Cell cycle analysis showed the ability of PA+HG to induce a G1 phase arrest (73.75% ± 4.57 vs. 54.24% ± 3.65 of Ctr, *p* < 0.001) accompanied by cyclin D1 and E1 protein accumulation (*p* < 0.001) (Figure 1F–I). Supplementation with 3-kDa WH counteracted the negative effects of PA+HG, decreasing the EC rate in the G1 phase (50.71% ± 6.26, *p* < 0.01), as the cyclin D1 and E1 upregulation (*p* < 0.01 vs. PA+HG) (Figure 1F–I).

### 3.3. WH Effects on PA+HG-Induced Apoptosis

**Figure 2:**
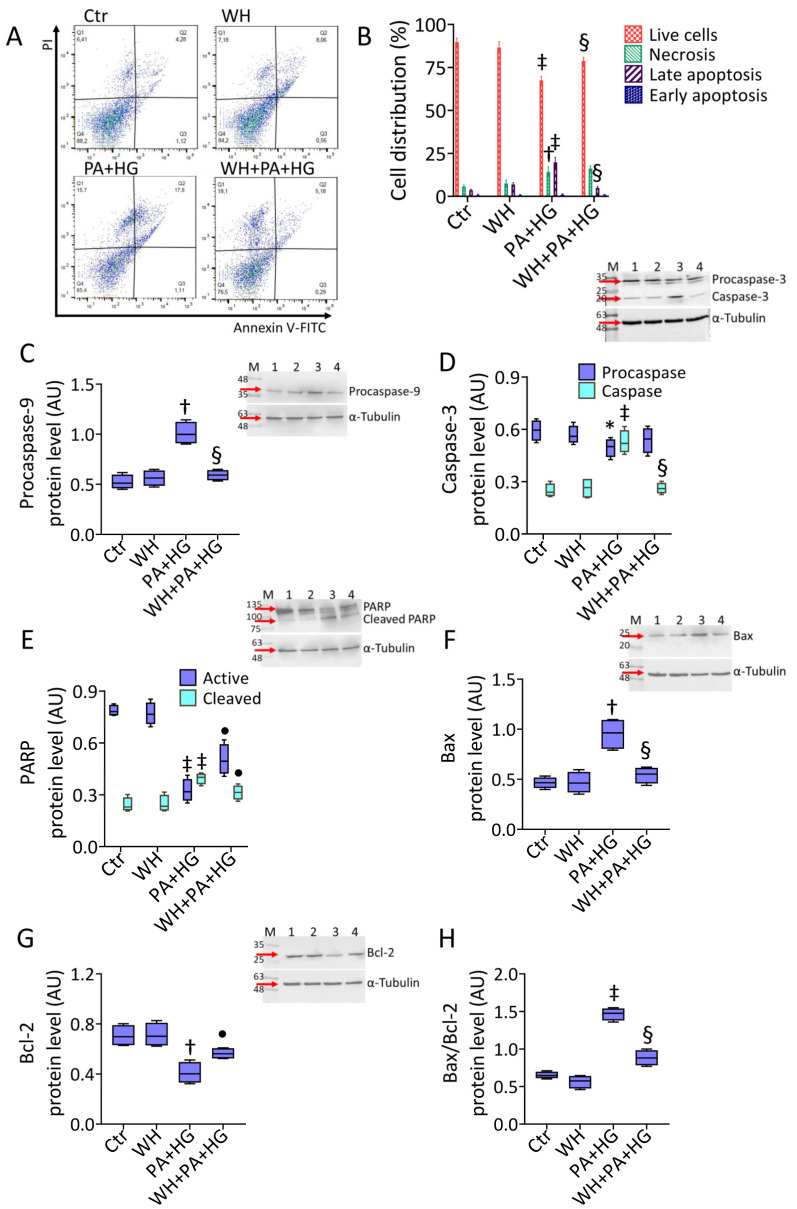
Whey reduced the PA+HG-mediated apoptosis. (**A**,**B**) Representative FACS analysis of annexin V-FITC and PI-staining and immunoblotting analysis with cropped blots of (**C**) procaspase-9, (**D**) caspase-3, (**E**) PARP, (**F**) Bax, (**G**) Bcl-2, and (**H**) Bax/Bcl-2 ratio. Q1: necrotic cells; Q2: late apoptotic cells; Q3: early apoptotic cells; Q4: viable cells. Data are reported as mean ± SD of n = 3 independent experiments. M = molecular weight markers, lane 1 = Ctr, lane 2 = WH, Lane 3 = PA+HG, lane 4 = WH+PA+HG. Western blotting results are expressed as arbitrary units (AU). * *p* < 0.05 vs. Ctr; † *p* < 0.01 vs. Ctr; ‡ *p* < 0.001 vs. Ctr; • *p* < 0.05 vs. PA+HG; § *p* < 0.01 vs. PA+HG.

The ability of 3-kDa WH to counteract the apoptotic mechanism triggered by exposure to PA+HG was then evaluated (Figure 2). The results indicated that 3-kDa WH supplementation attenuated the PA+HG-related decrease in live cells (78.73% ± 2.23 vs. 67.42% ± 2.38 in PA+HG, *p* < 0.01), as well as prevented the late apoptotic population (4.91% ± 0.89 vs. 19.79% ± 2.92 in PA+HG, *p* < 0.01) (Figure 2A,B). In particular, 3-kDa WH counteracted procaspase-9, caspase-3, and cleaved PARP upregulation of PA+HG cells, as well as the degradation of the active form of PARP (*p* < 0.01 vs. PA+HG) (Figure 2C–E). The results evidenced the ability of 3-kDa WH to contrast Bax accumulation along with the Bcl-2 decrease triggered by PA+HG, thus resulting in the attenuated Bax/Bcl-2 ratio (*p* < 0.01 vs. PA+HG) (Figure 2F–H).

### 3.4. WH Prevented the PA+HG-Induced Oxidative Stress

**Figure 3:**
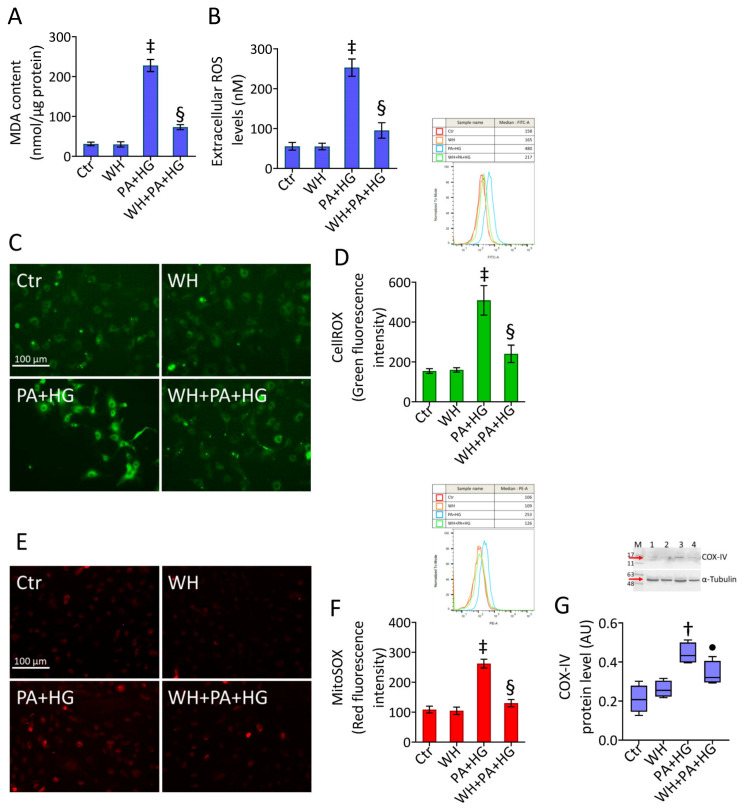
Whey prevented the PA+HG-related ROS accrual. (**A**) MDA content, (**B**) extracellular ROS levels and representative fluorescent images and FACS analysis of (**C**,**D**) intracellular and (**E**,**F**) mitochondrial ROS. Scale bars = 100 μm. (**G**) Immunoblotting analysis with cropped blots of COX-IV protein levels. Data are reported as mean ± SD of n = 3 independent experiments. M = molecular weight markers, lane 1 = Ctr, lane 2 = WH, Lane 3 = PA+HG, lane 4 = WH+PA+HG. Western blotting results are expressed as arbitrary units (AU). † *p* < 0.01 vs. Ctr; ‡ *p* < 0.001 vs. Ctr; • *p* < 0.05 vs. PA+HG; § *p* < 0.01 vs. PA+HG.

The evaluation of lipid peroxidation as well as the assessment of extracellular, intracellular, and mitochondrial ROS levels revealed that the extensive oxidative stress triggered by PA+HG (*p* < 0.001) was counteracted by 3-kDa WH (*p* < 0.01 vs. PA+HG) (Figure 3A–F). Treatment with the 3-kDa WH also abolished the COX-IV protein level accumulation (*p* < 0.05 vs. PA+HG) (Figure 3G).

### 3.5. Mitochondrial Dysfunction in T2DM-EC

**Figure 4:**
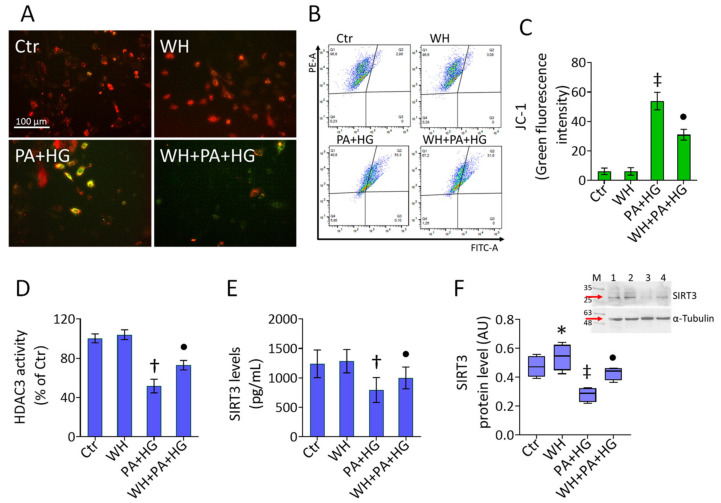
Whey ameliorated the PA+HG-induced mitochondrial damage. (**A**–**C**) Representative fluorescent images and FACS analysis of mitochondrial membrane potential and (**D**) HDAC3 activity. SIRT3 levels evaluated by (**E**) ELISA kit and (**F**) immunoblotting analysis. Scale bars = 100 μm. Data are reported as mean ± SD of *n* = 3 independent experiments. M = molecular weight markers, lane 1 = Ctr, lane 2 = WH, Lane 3 = PA+HG, lane 4 = WH+PA+HG. Western blotting results are expressed as arbitrary units (AU). * *p* < 0.05 vs. Ctr; † *p* < 0.01 vs. Ctr; ‡ *p* < 0.001 vs. Ctr; • *p* < 0.05 vs. PA+HG.

JC-1 dye staining of T2DM EC showed that mitochondria had significantly low membrane potential during exposure to PA+HG (*p* < 0.01). The detrimental depolarization was counteracted by 3-kDa WH (*p* < 0.05 vs. PA+HG) (Figure 4A–C). The mitophagy assay revealed the capacity of 3-kDa WH to prevent both functional mitochondrial decrease and lysosome accumulation in PA+HG cells (*p* < 0.05 vs. PA+HG) (Figure S2). The PA+HG-mediated mitochondrial injury led to the downregulation of HDAC3 activity (*p* < 0.01) along with decreased SIRT3 protein levels (*p* < 0.001), phenomena abolished by WH supplementation (*p* < 0.05 vs. PA+HG) (Figure 4D–F).

### 3.6. WH Effects on PA+HG-Mediated Mitochondrial Metabolic Dysfunction 

**Figure 5:**
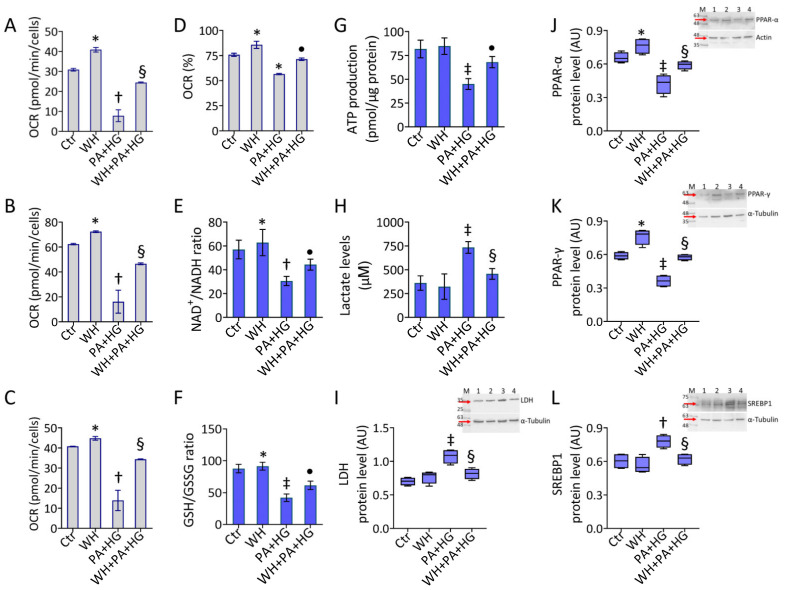
Whey beneficial effects on mitochondrial and metabolic homeostasis. (**A**) ATP production coupled respiration, (**B**) maximal and (**C**) basal respiration, and (**D**) coupling efficiency assessed by Seahorse analyzer. (**E**) NAD^+^/NADH and (**F**) GSH/GSSG ratios, (**G**) ATP levels, (**H**) lactate content and immunoblotting analysis with cropped blots of (**I**) LDH, (**J**) PPAR-α, (**K**) PPAR-γ, and (**L**) SREBP1. Data are reported as mean ± SD of n = 3 independent experiments. M = molecular weight markers, lane 1 = Ctr, lane 2 = WH, Lane 3 = PA+HG, lane 4 = WH+PA+HG. Western blotting results are expressed as arbitrary units (AU). * *p* < 0.05 vs. Ctr; † *p* < 0.01 vs. Ctr; ‡ *p* < 0.001 vs. Ctr; • *p* < 0.05 vs. PA+HG; § *p* < 0.01 vs. PA+HG.

To deepen the effects elicited by 3-kDa WH on cell metabolism and PA+HG-induced endothelial damage, mitochondrial respiration and glycolytic state were investigated (Figure 5). Treatment with 3-kDa WH improved the endothelial ATP production coupled respiration (*p* < 0.01), the maximal and basal respiration (*p* < 0.05), and the percentage of coupling efficiency (*p* < 0.05) compared to the control cells (Figure 5A–D and Figure S3). Of note, 3-kDa WH prevented the negative effects exerted by PA+HG, increasing the mitochondrial respiration efficiency (*p* < 0.01 vs. PA+HG) (Figure 5A–D and Figure S3). Furthermore, 3-kDa WH enhanced the NAD^+^/NADH and GSH/GSSG ratios (*p* < 0.05), counteracted the PA+HG-mediated downregulation (*p* < 0.05 vs. PA+HG) (Figure 5E,F), and prevented ATP depletion (*p* < 0.05 vs. PA+HG) and LDH accumulation (*p* < 0.01 vs. PA+HG) (Figure 5G–I). Treatment with the 3-kDa WH displayed positive modulation on key metabolic proteins, PPAR-α (*p* < 0.05) and PPAR-γ (*p* < 0.01), opposing their reduction in PA+HG-treated cells (*p* < 0.01 vs. PA+HG) (Figure 5J,K). Finally, the reduction in the active SREBP1 protein expression (*p* < 0.01 vs. PA+HG) was also observed (Figure 5L).

### 3.7. SIRT3^-^ Prevented the WH Protective Effects on Mitochondrial Dysfunction

**Figure 6:**
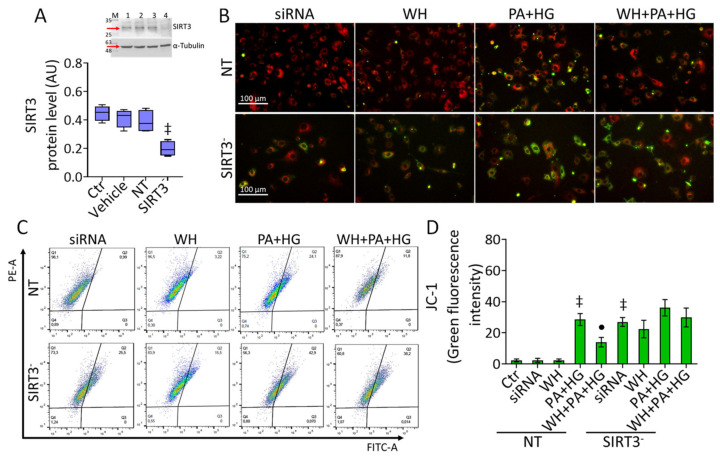
SIRT3 depletion abolished the protective effects of whey. (**A**) Representative immunoblotting analysis with cropped blot of SIRT3 protein levels in EC treated with empty transfection reagent (Vehicle) or transfected with nontargeting siRNA control (NT) or SIRT3 siRNA (SIRT3^−^). M = weight markers, lane 1 = Ctr, lane 2 = Vehicle, lane 3 = NT, lane 4 = SIRT3^-^. Western blotting data are expressed as arbitrary units (AU). (**B**–**D**) Representative fluorescent images and FACS analysis of mitochondrial membrane potential. Scale bars = 100 μm. Data are reported as mean ± SD of n = 3 independent experiments. ‡ *p* < 0.001 vs. NT; • *p* < 0.05 vs. NT+PA+HG.

Given the results concerning SIRT3 negative modulation and considering the critical role of this sirtuin in diabetes-associated oxidative stress and endothelial dysfunction [45], we explored whether SIRT3 silencing (*p* < 0.001 vs. NT) (Figure 6A) could affect the beneficial effects of 3-kDa WH on T2DM-induced stress. The results showed that the downregulation of SIRT3 triggered mitochondrial membrane depolarization (*p* < 0.001 vs. NT), aggravated the PA+HG detrimental action, and abolished the protective effects of WH against mitochondrial dysfunction Figure 6B–D and Figure S4).

### 3.8. SIRT3^-^ Abolished the Beneficial Ability of WH against Oxidative Stress

**Figure 7:**
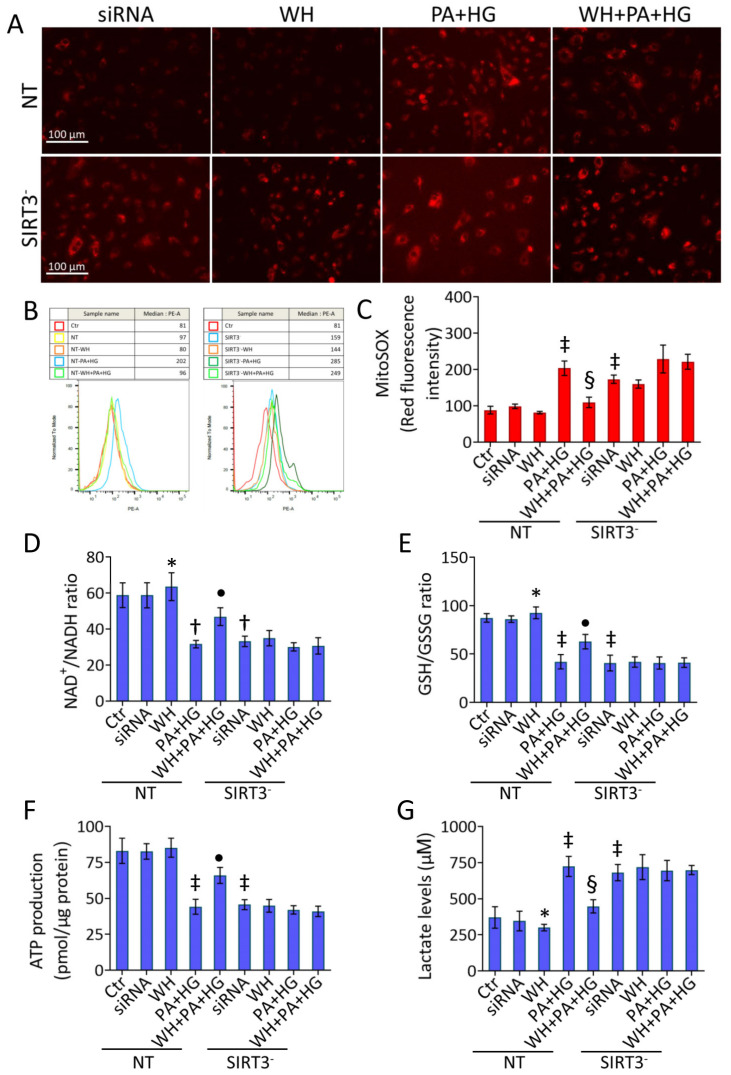
SIRT3 silencing suppressed the whey beneficial effects on metabolic pathways. (**A**–**C**) Representative fluorescent images and FACS analysis of mitochondrial ROS. Scale bars = 100 μm. Detection of (**D**) NAD^+^/NADH and (**E**) GSH/GSSG ratios, (**F**) ATP levels, and (**G**) lactate content. Data are reported as mean ± SD of *n* = 3 independent experiments. * *p* < 0.05 vs. NT; † *p* < 0.01 vs. NT; ‡ *p* < 0.001 vs. NT; • *p* < 0.05 vs. NT+PA+HG; § *p* < 0.01 vs. NT+PA+HG.

Analysis of mitochondrial ROS revealed the SIRT3^-^ capacity to target ROS accumulation (*p* < 0.001 vs. NT) and to exacerbate the negative effects of PA+HG stress, blunting the opposing ability of 3-kDa WH (Figure 7A–C and Figure S5). Similarly, SIRT3 deprivation affected the cellular redox metabolism by decreasing the ATP content, NAD^+^/NADH and GSH/GSSG ratios, and inducing LDH accrual (*p* < 0.001 vs. NT), withdrawing the protective effects exerted by 3-kDa WH supplementation (Figure 7D–G).

## 4. Discussion

Herein, we report first evidence on the protective role of whey in dysfunctional endothelium by ameliorating the cytotoxic effects of the T2DM milieu, mimicked by the combined PA+HG stress. The results from this study showed the ability of 3-kDa whey fraction, containing considerable amounts of betaines and short-chain acylcarnitines, to ameliorate the endothelial cytotoxicity induced by combined hyperlipidic (0.1 mM PA) and hyperglycemic (30 mM HG) stress, and to prevent the cycle arrest in the G1 phase and the intrinsic apoptosis sustained by caspase-3 activation. 

Chronic hyperglycemia and hyperlipidemia can directly result in the injury of EC, a key initiator of diabetic cardiovascular events [46]. Endothelial abnormalities act as a determining factor in chronic diabetes complications, leading to aberrant ROS production, oxidative stress, and dysregulated inflammation [47]. Indeed, in diabetic patients, EC exhibit functional alterations in mitochondria and reduced oxidative ability [48]. In response to hyperglycemia and hyperlipidemia, the mitochondria-related ROS generation acts as a common upstream event, initiating a plethora of responses converging into multiple pathogenic mechanisms as autophagy, NADPH-oxidases, and uncoupled endothelial nitric oxide synthase (eNOS) induction, causing nuclear DNA double-strand breaks, PARP activation, and apoptotic mechanisms [13,49]. Our study revealed that whey supplementation alleviated the diabetic mitochondrial dysfunction in EC and downregulated apoptotic cell death. In addition, whey ameliorated endothelial redox impairment and reduced lipidic peroxidation, extracellular, intracellular, and mitochondrial ROS accumulation under PA+HG stimulation.

The great interest in natural sources of antioxidants able to enhance antioxidant mechanisms and protect EC from the harmful effects of oxidative stress has been reported and several clinical trials targeting oxidative damage have been conducted for T2DM and its complications [50]. The health benefits of dairy foods could be related to oxidative stress and low-grade systemic inflammation reduction providing an inverse association between total dairy and low-fat dairy consumption and T2DM [51]. However, the impact of dairy products remains controversial. The deep comprehension and the underlying mechanisms triggered by food in regulating glucose and lipid metabolism, as well as diet influence on genetic and epigenetic pathways result critical for developing novel preventive and therapeutic approaches for T2DM [52,53]. Several studies discussing the relationship between food and T2DM risk reported conflicting evidence [54,55]. Whey protein is a widely consumed supplement able to increase the antioxidant defense [56]. Despite being a by-product of cheese manufacturing, it is used as a functional food with nutritional applications [57]. Dietary supplementation with whey protein has been shown to improve blood pressure, systemic vascular resistance, and arterial stiffness in overweight and obese subjects [58]. Moreover, an improvement in microvascular function has been observed after long-term supplementation with whey protein, compared to placebo, in patients with heart failure [59]. The ingestion of whey protein before/after intense exercise enhanced insulin-stimulated perfusion in T2DM individuals [60]. Milk-derived whey ameliorated insulin secretion in obese, prediabetic, and T2DM subjects, reducing postprandial hyperglycemia and improving lipidemia by exerting antioxidative effects, as an enhancer of GSH synthesis and endogenous antioxidative enzyme system [61]. Similarly, the present study showed that 3-kDa whey fraction was effective in counteracting GSH/GSSG and NAD^+^/NADH depletion and able to protect EC from oxidative stress-induced damage by regulating key antioxidant and metabolic enzymes such as LDH, PPAR-α, PPAR-γ, and SREBP1. In addition, our results revealed the ability of whey to prevent the loss of mitochondrial membrane potential, along with an accumulation of damaged mitochondria and lysosomes, thus suggesting whey as a possible “mitochondrial nutrient” that can preserve mitochondrial function during metabolic diseases, such as T2DM. Our data highlight that the functional properties of 3-kDa whey fraction to counteract T2DM-related mitochondrial and metabolic injury and to preserve endothelial homeostasis can be ascribed to the notable content of betaines, l-carnitine, and acylcarnitines and to the synergism among these biomolecules [32]. These bioactive compounds, such as betaines and short-chain acylcarnitines, are pivotal in human health and metabolically important in displaying anti-inflammatory and antioxidant properties [62,63]. Therefore, the search for novel dietary sources highly rich in specific nutrients with health-promoting properties is still an open door.

The role of peroxisome proliferator-activated receptors (PPARs) in T2DM is well known, since they control the expression of genes related to lipid and glucose homeostasis [64]. In particular, PPAR-α and PPAR-γ, ligand-activated transcription factors, regulate glucose, fatty acid, and lipid metabolism, inflammation and, especially in the pathogenesis of obesity, hypercholesterolemia, insulin resistance, and atherosclerosis [65]. To date, PPARs represent an attractive link between genes and environmental stimuli, being activated by a variety of environmental variables, including dietary components [66]. In this context, several reports described the role of natural products in targeting PPAR and its impact on cardiovascular events [67]. We showed that the 3-kDa whey ameliorated the PA+HG-related LDH and SREBP1 accrual, as well as PPAR-α and PPAR-γ protein level reduction, suggesting that it could serve as a potential therapeutic strategy in T2DM for targeting PPAR-α and PPAR-γ.

SIRT3 is a critical regulator of glucose flux under conditions of nutrient overload, as systemic insulin sensitivity and insulin-stimulated muscle glucose uptake are significantly impaired in the absence of SIRT3 [68]. Reduced SIRT3 levels have been related to decreased viability in EC isolated from diabetic patients, further confirmed by in vitro low viability in SIRT3-silenced EC exposed to hyperglycemia [69]. Lower serum SIRT3 levels have been detected in T2DM patients with coronary artery disease than in those without coronary injury [70]. Similarly, reduced SIRT3 protein levels were unveiled in both the culture media and cell homogenate of EC exposed to PA+HG. Of interest, the results of this study showed the effect of whey to prevent the SIRT3 downregulation induced by PA+HG stress. In vitro and in vivo studies reported the effects of antioxidant agents to improve the endothelium state and inhibit ROS production by targeting SIRT3, with impaired protective effects during the SIRT3 repression [71,72]. Likewise, our data showed that SIRT3 deficiency exacerbated the PA+HG-mediated cytotoxicity and abolished the beneficial outcome exerted by whey on endothelial damages induced by PA+HG, thus demonstrating that the protective effects might be associated, at least in part, with its capacity to modulate SIRT3. Therapeutic strategies targeting post-translational modification of this protein have shown a pivotal potential in the treatment of diabetes, as well as showing the modulation of oxidative stress via SIRT3 has clearly been effective in this multifaceted disease [73]. Nowadays, the role of mitochondrial-targeting antioxidants able to diminish oxidative stress, alleviating vascular prognosis in patients with diabetes, is increasingly recognized [74,75], although multiple aspects still warrant further investigations. To date, the potential health benefits of whey in T2DM models are still little explored and additional future in-depth in vitro and in vivo studies on the role of each biomolecule and the complex interactions among them are critical to support the health potential of whey in the diabetic state.

## 5. Conclusions

Mitochondrial metabolism is a critical hallmark of dysfunctional endothelium during T2DM. Whey, rich in bioactive components and functional activities, preserved endothelial cells from PA+HG-induced cytotoxicity, apoptosis, ROS accumulation, impaired mitochondria, and altered redox metabolic pathways. The protective effects exerted by whey upregulated SIRT3 protein levels in the presence of PA+HG. SIRT3 depletion blocked the effects of whey, suggesting SIRT3 as a potential key molecular player in the biochemical pathway activated by whey. In the perspective of circular economy and environmental sustainability, these observations underline the importance of considering whey, rather than a by-product, as a food source of biomolecules for use in the development of innovative preventive approaches in the diabetic state.

## Figures and Tables

**Table 1:** Functional biomolecule levels (mg/L) in whey evaluated by HPLC-ESI-MS/MS analysis.

Bioactive Compound	*m*/*z*	MS/MS Transition	Levels (mg/L)
l-Carnitine	162.1	162.1 → 103	43.4 ± 1.6
Acetyl-l-carnitine	204.1	204.1 → 85	6.5 ± 3.1
Propionyl- L-carnitine	218.1	218.1 → 85	29.4 ± 2.2
Glycine betaine	118.1	118.1 → 59	13.2 ± 1.2
δ-valerobetaine	160.1	160.1 → 101	26.7 ± 2.3
γ-butyrobetaine	146.1	146.1 → 87	6.8 ± 0.9

## Data Availability

The data presented in this study are available from the corresponding author upon request.

## Supplementary Materials

The following supporting information can be downloaded at https://www.mdpi.com/article/10.3390/antiox12061311/s1, Figure S1: Dose- and time-response of PA on HG cells; Figure S2: Whey effects on PA+HG-induced mitophagy; Figure S3: Whey effects on mitochondrial respiration; Figure S4: Mitochondrial membrane polarization controls; Figure S5: Mitochondrial ROS controls.
