# Supplementary material for: Whey Improves In Vitro Endothelial Mitochondrial Function and Metabolic Redox Status in Diabetic State
Source: Antioxidants (Basel). 2023 Jun 20;12(6):1311. doi: 10.3390/antiox12061311 (PMC10295385; doi:10.3390/antiox12061311)
Supplement: Supplementary file 1 [file antioxidants-12-01311-s001.zip › antioxidants-2452244-supplementary.pdf]

Whey improves in vitro endothelial mitochondrial function and metabolic redox status in diabetic state

Elisa Martino[1,†], Amalia Luce[1,†], Anna Balestrieri[2], Luigi Mele[3], Camilla Anastasio[1], Nunzia D'Onofrio[1], Maria Luisa Balestrieri[1,*], Giuseppe Campanile[4]

[1] Department of Precision Medicine, University of Campania Luigi Vanvitelli, Via L. De Crecchio 7, 80138 Naples, Italy; elisa.martino@unicampania.it (E.M.), amalia.luce@unicampania.it (A.L.), camilla.anastasio@unicampania.it (C.A.), nunzia.donofrio@unicampania.it (N.D.).

[2] Food Safety Department, Istituto Zooprofilattico Sperimentale del Mezzogiorno, Portici, Italy; anna.balestrieri@izsmportici.it (A.B.).

[3] Department of Experimental Medicine, University of Campania Luigi Vanvitelli, Via Luciano Armanni 5, 80138 Naples, Italy; luigi.mele@unicampania.it (L.M.).

[4] Department of Veterinary Medicine and Animal Production, University of Naples Federico II, Naples, Italy; giuseppe.campanile@unina.it (G.C.).

* Correspondence: marialuisa.balestrieri@unicampania.it; Tel.: +39-081-566-5865

† These authors contributed equally to this work.

SUPPLEMENTARY MATERIALS

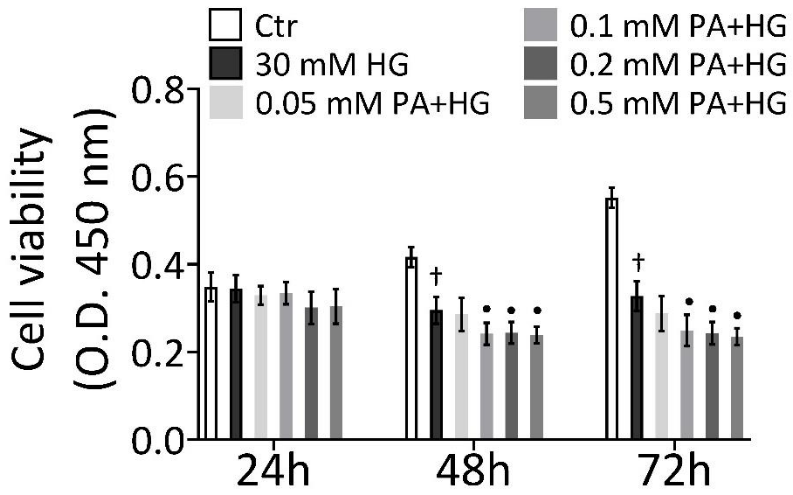

Figure S1. *Dose- and time-response of PA on HG-cells*. EC viability following exposure for 24, 48 and 72h to different doses of PA in EC stimulated with HG (30mM). Data are reported as mean ± SD of n = 3 independent experiments. †p<0.01 vs. Ctr; •p<0.05 vs. HG.

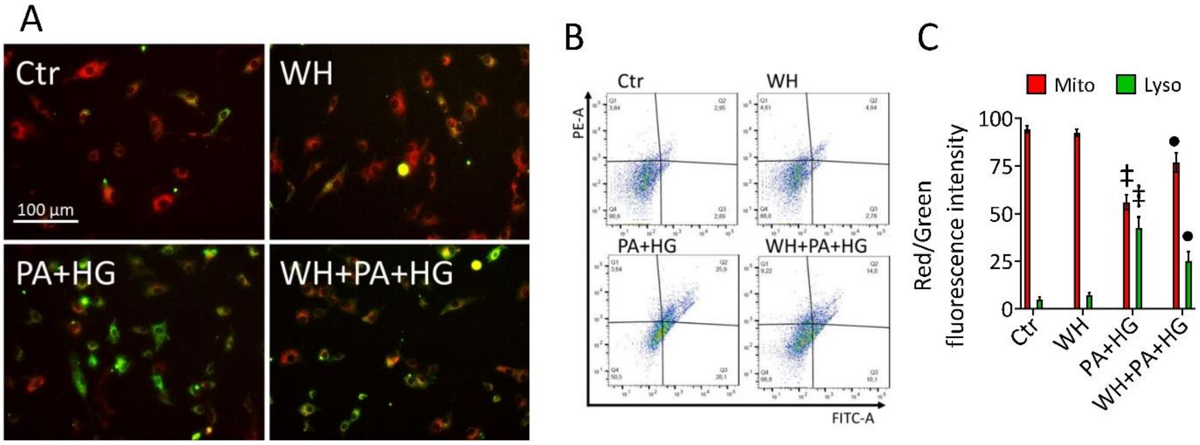

Figure S2. *Whey effects on PA+HG-induced mitophagy*. (A) Representative fluorescent images and (B,C) FACS analysis of intact mitochondria (red) and lysosome (green) stains. Scale bars = 100 μm. Data are reported as mean ± SD of n = 3 independent experiments. ‡p<0.001 vs. Ctr; •p<0.05 vs. PA+HG.

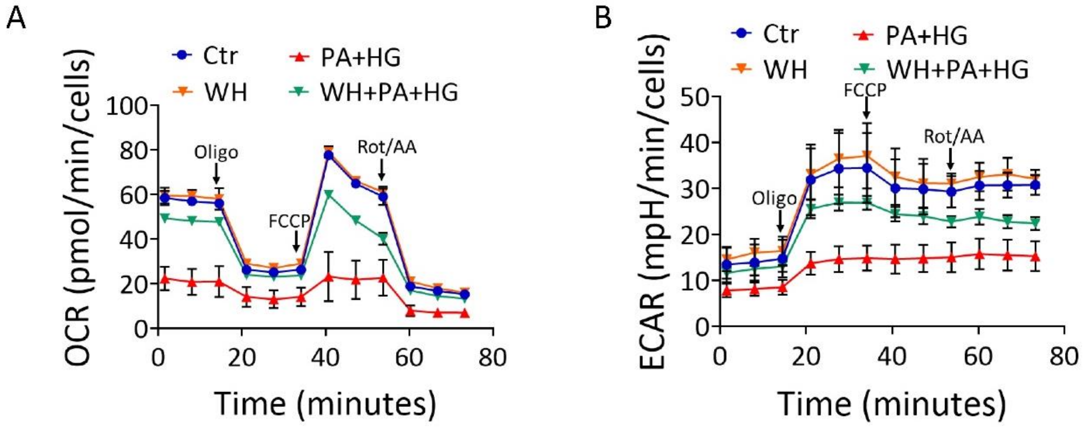

Figure S3. *Whey effects on mitochondrial respiration*. (A) Oxygen consumption rate (OCR) and (B) extracellular acetylation rate (ECAR) assessed with Seahorse analyzer in EC treated cells.

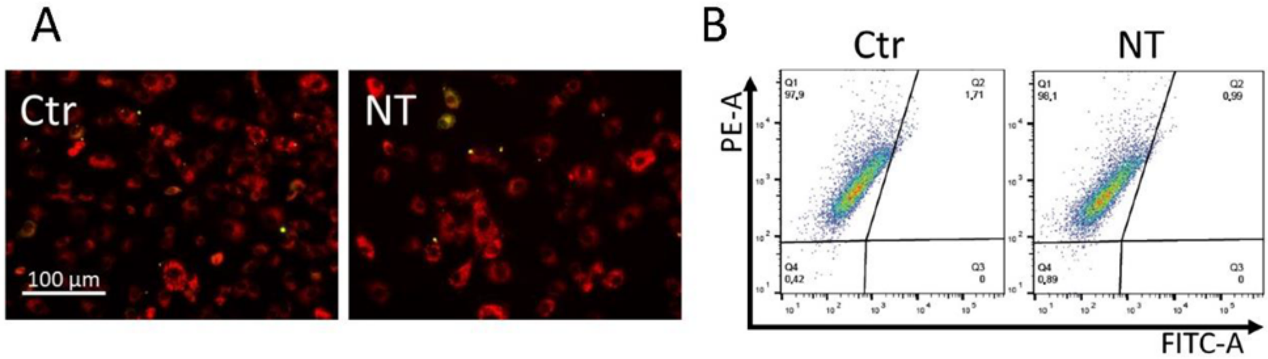

Figure S4. *Mitochondrial membrane polarization controls.* (A) Representative fluorescent images and (B) FACS analysis of mitochondrial membrane potential of EC transfected with non-targeting siRNA control (NT) or not transfected (Ctr). Scale bars = 100 µm.

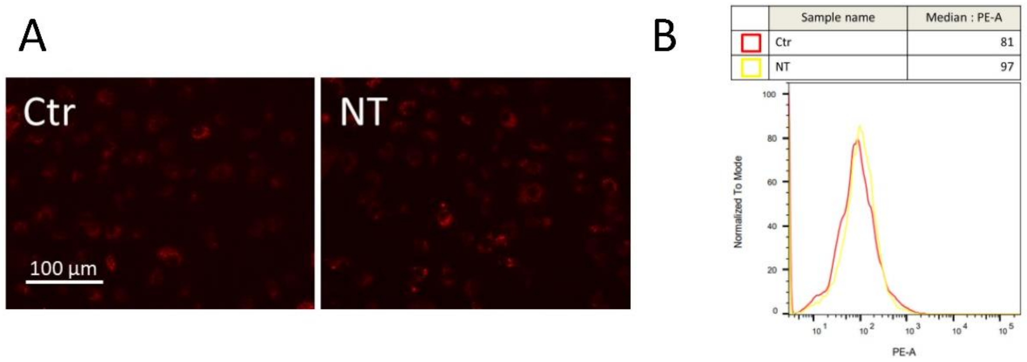

Figure S5. *Mitochondrial ROS controls*. (A) Representative fluorescent images and (B) FACS analysis of mitochondrial ROS levels assessed in EC transfected with non-targeting siRNA control (NT) or not transfected (Ctr). Scale bars = 100 μm.
